# Health workers’ performance in the implementation of Patient Centred Tuberculosis Treatment (PCT) strategy under programmatic conditions in Tanzania: a cross sectional study

**DOI:** 10.1186/1472-6963-13-101

**Published:** 2013-03-16

**Authors:** Abdallah Mkopi, Nyagosya Range, Mbaraka Amuri, Eveline Geubbels, Fred Lwilla, Saidi Egwaga, Alexander Schulze, Frank van Leth

**Affiliations:** 1 Ifakara Health Institute, P.O. Box 78373, Dar es Salaam, Tanzania; 2 National Institute for Medical Research, Dar es Salaam, Tanzania; 3 National Tuberculosis and Leprosy Programme, Ministry of Health and Social Welfare, Dar es Salaam, Tanzania; 4 Novartis Foundation for Sustainable Development, Basel, Switzerland; 5 KNCV Tuberculosis Foundation, The Hague, The Netherlands; 6 Department of Global Health, Academic Medical Centre, Amsterdam Institute for Global Health and Development, University of Amsterdam, Amsterdam, The Netherlands

**Keywords:** TB, Directly observed treatment, PCT, Home-based, Patient centred treatment, Tanzania

## Abstract

**Background:**

Patient Centred Tuberculosis Treatment (PCT) is a promising treatment delivery strategy for Mycobacterium tuberculosis (TB). It aims to improve adherence to treatment by giving patients the choice of having drug intake supervised at the health facility by a medical professional or at home by a supporter of their choice.

**Methods:**

A cross-sectional survey was undertaken in three districts of Tanzania during October 2007, one year after PCT was rolled out nationally. Semi-structured questionnaires were used to assess whether key elements of the PCT approach were being implemented, to evaluate supporters’ knowledge, to capture opinions on factors contributing to treatment completion, and to assess how treatment completion was measured. Transcripts from open-ended responses were analysed using framework analysis.

**Results:**

Interviews were conducted with 127 TB patients, 107 treatment supporters and 70 health workers. In total, 25.2% of TB patients were not given a choice about the place of treatment by health workers, and only 13.7% of those given a choice reported that they were given adequate time to make their decision. Only 24.3% of treatment supporters confirmed that they were instructed how to complete patients’ treatment cards. Proper health education was the factor most frequently reported by health workers as favouring successful completion of TB treatment (45.7%). The majority of health workers (68.6%) said they checked returned blister packs to verify whether patients had taken their treatment, but only 20.0% checked patients’ treatment cards.

**Conclusions:**

The provision of choice of treatment location, information on treatment, and guidance for treatment supporters need to be improved. There is a requirement for regular re-training of health workers with effective supportive supervision if successful implementation of the PCT approach is to be sustained.

## Background

Patient Centred Tuberculosis Treatment (PCT) is a promising treatment delivery strategy for *Mycobacterium tuberculosis* (TB). Globally, TB killed 4,700 people per day in 2009. In Tanzania, 63,450 cases of all forms of TB were recorded in 2010, ranking it 16^th^ among the 22 countries that have the highest TB burden worldwide [1].

TB has become an increasingly serious problem in countries with a high prevalence of HIV, since HIV-positive patients are more susceptible to infection [2-5]. The World Health Organization changed its recommendations regarding first-line therapy for TB in an attempt to address the higher rate of relapse in HIV-positive patients in whom TB was managed with a regimen that included only two months’ treatment with rifampicin. A first-line treatment regimen that includes rifampicin throughout the entire duration of treatment is preferred [6]. Against this background, Tanzania introduced Fixed-Dose Combination therapy (FDC) in 2006, in which rifampicin is administrated throughout the full six-month treatment period [1]. Given the pivotal role that rifampicin plays in TB management, the current TB control strategy recommends strict observation of its intake. In order to ensure adequate supervision of rifampicin administration during the six-month treatment period, Tanzania introduced an innovative approach called Patient Centred Treatment (PCT) in 2006. The primary goal of the PCT approach is to enhance access and adherence to TB therapy by allowing TB patients to choose where rifampicin intake is supervised and by whom. Supervision can be undertaken by a medical professional at a health facility, or at home by a supporter of the patient’s choice. In both cases, Directly Observed Treatment (DOT) is respected. Patients (or their supporter if the patient is too unwell) collect their drugs each week during the intensive phase (the first two months) then every two weeks during the continuation phase (the following four months). PCT reduces the burden on health workers since they do not have to supervise the drug intake of all TB patients every day, an important factor in view of the growing number of TB patients in a setting with a chronic shortage of health workers. Moreover, the conventional DOT approach, by which patients were obliged to attend the health facility each day to obtain medication, was difficult for many patients because of their poor physical condition, the distances involved, and lack of funds to pay for travel [7]. Over-worked healthcare teams could result in suboptimal patient care, while barriers to daily attendance at a health facility increase the risk of non-adherence to the treatment regimen. A needs assessment undertaken before implementing PCT confirmed that it was regarded as a positive aspect of the national TB control strategy by both patients and health care workers.

Within the PCT strategy, the patient is passively identified. The time period between becoming aware of symptoms and visiting a health facility is not influenced. Thus, access to healthcare is not improved by the PCT approach *per se*. PCT does, however, enable health workers to focus on the management of newly diagnosed TB patients instead of being overwhelmed by DOT activity for all patients under their care. This could be expected to contribute to improved assessment and diagnostic procedures, such that PCT may indirectly support improved access to diagnosis and, if required, treatment. The key advantage of PCT for the patient lies with the choice of treatment location and supporter, This empowerment of the patient for an important part of his/her treatment can positively influence treatment adherence. Proper diagnosis, treatment and adherence are the cornerstones of effective TB control.

The effectiveness of the PCT approach was assessed in three pilot districts. It showed positive effects on treatment outcome, with most treatment supporters being family members [8]. A study in Nepal documented that family members of TB patients can help to ensure adherence to treatment just as successfully as community health workers [9]. Moreover, an earlier systematic review of different types of DOT showed that DOT with multiple treatment supporters showed higher rates of treatment completion than strategies using only conventional health facility-based DOT or unsupervised TB treatment [10]. Typical patient enablers included home-based support [11].

All aspects of PCT should be implemented consistently across health facilities if sustainable scale-up is to be achieved. In order to identify areas where implementation might pose difficulties, we assessed health workers’ performance in delivering PCT one year after the introduction of PCT. Performance was assessed through interviews with patients, health workers and treatment supporters. The aim of the study was to gather evidence on operational aspects of the PCT approach which can inform TB control policy and service delivery in Tanzania.

## Methods

### Setting and study population

The study was designed as a cross-sectional survey with a single four-week period of data collection during October 2007. One year prior to the survey, the PCT strategy had been scaled up nationally by introducing new guidelines with minimal extra training or supervision.

The study was carried out in the Arusha Municipality, Kahama and Mufindi districts of Tanzania, where the PCT approach was piloted. The selection criteria for the study sites have been described in detail elsewhere [8]. Arusha Municipality is an urban site while Mufindi and Kahama are rural districts. Arusha Municipality is one of five districts in the Arusha region in Tanzania. TB care is provided in 19 health facilities, of which six are hospitals, four are health centres, and nine are dispensaries. Mufindi district in the Iringa region has a total of 36 health facilities for TB care (two hospitals, six health centres and 28 dispensaries). Mufindi is predominantly occupied by the Hehe, Bena and Kinga tribes, each of which has its distinct local language, local beliefs and traditional lifestyles. Kahama district is located in the Shinyanga region in north-western Tanzania, where the major ethnic group is the Sukuma tribe. There are 35 health facilities providing TB care (one hospital, four health centres and 30 dispensaries).

The study population consisted of newly registered TB patients who were receiving TB treatment, their treatment supporters, and health workers in the facilities where the patients were registered. In each district, half of the TB diagnostic centres were randomly sampled using a proportional-to-size strategy based on the number of new TB patients registered. The sampling frame was based on 2006 notification data obtained from the National TB and Leprosy Programme (NTLP). Within each sampled centre, the most recently registered TB patients were to be included until the specified number of patients for that district had been reached, with a planned total of 120 patients (50 in Arusha, 40 in Mufindi and 30 in Kahama). The varying numbers reflected differences in the number of cases notified in each district. Patients were eligible for inclusion if they had not received TB treatment for more than a month prior to the study and were currently receiving TB treatment either at home or at the health facility. When selected patients could not be contacted or declined to participate, the next most recently registered patient at the same health facility was approached. All treatment supporters for participating patients were invited to take part. For each health facility at least two healthcare providers who were directly involved in TB service provision and who were on duty on the day the data collection team were present were selected to participate. In facilities where only one healthcare worker was present (mainly dispensaries), this person was included in the study.

### Data collection

Data were collected during face-to-face interviews using semi-structured questionnaires. All questionnaires were designed in English and translated into Swahili, with back-translation into English to ensure proper translation. During the interviews information was gathered on the personal characteristics of the patient (age, sex), current TB treatment (start date, type of DOT), the patient’s choice within PCT, relationship with the chosen treatment supporter, the patient’s opinion of PCT, and healthcare providers’ experience with TB and its treatment.

Key elements of the PCT approach were also investigated. The key aspect is that health workers have to inform all TB patients that they have a choice with regards to the place of treatment. Health workers are also supposed to give patients enough time (at least up to two weeks) to decide on the treatment location and to identify a treatment supporter of their choice. The study assessed if adequate choice was given to patients, that is, assessing if health workers gave patients at least up to two weeks for decision-making as stated in the PCT guideline [12]. When patients were offered the choice and given enough time to decide, the outcome was classified as ‘adequate’. Additionally, the survey evaluated whether health workers explained potential side effects to patients. Health workers are supposed to alert patients to possible minor side effects (including loss of appetite, nausea, abdominal pain, joint pains, burning sensation in feet and orange/red urine) and major side effects (itching of skin, skin rash, deafness, dizziness/lack of balance, jaundice repeated vomiting and difficulty with vision).

Treatment supporters were asked about their activities with regards to care of TB patients. According to the PCT guideline, treatment supporters are requested to collect the drugs at the health facility, ensure that the patient takes the drugs as prescribed by observing intake, and keep records of the daily drug intake on the patients’ treatment cards [12]. The survey also asked if treatment supporters had been instructed to fill out patients’ treatment cards and monitor patients’ adherence.

Among the health workers, the survey assessed which factors they identified as being instrumental in treatment completion, as well as the methods used to assess treatment completion.

All participating patients and their treatment supporters were invited to their respective health facilities. Interviews were conducted at the health facilities except in a few instances where patients had to be visited at home due to their serious condition or lack of transport. Interviews with patients, treatment supporters and health workers were carried out separately to allow each party to speak freely with the interviewer. Interviews were conducted in the local language. In the urban Arusha district, Kiswahili was used throughout. In the rural districts of Kahama and Mufindi the dominant local languages (Kisukuma and Kihehe respectively) were used if respondents were not able to express themselves fluently in Kiswahili. The majority of people in the two rural study sites are illiterate. Health workers who were not involved in TB treatment and care, or community members, served as interpreters and back-translation was performed on the spot.

Twelve field interviewers were trained for three days in September 2007. Soon after training, the tools were pre-tested for one day in order to check the flow and relevance of the questions, and to estimate the duration of each interview. In each district, the study team consisted of four interviewers and one research scientist.

### Data processing and analysis

Answers to open-ended questions in the semi-structured questionnaires were tape-recorded, transcribed in the local language and then translated into English. All transcripts were saved in Rich Text Format (RTF) and then imported into MAXQDA software (Udo Kuckartz, Berlin, Germany) for qualitative analysis. The qualitative analysis was undertaken using a thematic framework [13] which enabled identification of all key issues and themes by which the data could be examined. Open-ended answers were post-coded by creating different variables for each question. Data on baseline characteristics and post-coded variables were double-entered by two independent data entry clerks. Multiple answers for some variables per participant were possible. The descriptive statistics were performed using STATA software (StataCorp L, Texas 77845, USA).

### Ethical approval

The study was approved by the Institutional Review Board of the Ifakara Health Institute in Tanzania. Verbal consent was obtained from all participants, since the high illiteracy rate in two of the three study sites made written consent impractical. Researchers explained the study purpose to patients and explained that they could choose not to be interviewed without repercussions on them or to the continuation of treatment. The ethical review board waived the need for written consent in this study.

## Results

### Baseline characteristics

Of the 90 health facilities in the three districts, 45 were sampled (Arusha 10/19, Mufindi 17/35, Kahama 18/36). Interviews were conducted with 127 TB patients, 107 treatment supporters and 70 health workers. In addition to the planned 120 patients, a further seven patients were interviewed because all patients who presented at the health facilities on the day of data collection were included, such that the total number of patients was 127. Not all identified treatment supporters could be reached, resulting in a participation rate of 89.2% (107/120). The participation rate of healthcare providers was lower than expected (70/90, 77.8%) because it was not possible to identify two healthcare providers who provided TB services at each facility as had been planned. Moreover, some of the facilities, especially dispensaries, were staffed with only one relevant healthcare worker.

Among the 127 TB patients interviewed, 64.6% (n = 82) were male (Table 1). The median age of patients was 34.6 years (interquartile range [IQR]: 20 years) and 74.0% (n = 94) had completed at least primary education. Of the 107 treatment supporters, 57.9% (n = 62) were female, 63.5% (n = 68) had completed primary school and the median age was 36 years (IQR: 18 years). A majority of the 70 health workers were male (61.4%); their qualifications are summarised in Table 1.

**Table 1:** Characteristics of the study participants

	**Arusha**	**Kahama**	**Mufindi**	**Total**
**TB patients**	**47**	**34**	**46**	**127**
Male (n, %)	30 (63.8)	25 (73.5)	27 (58.7)	82 (64.6)
Median age (years) IQR	33	35	36	34.6
*Education*				
None (n, %)	6 (12.8)	8 (23.5)	4 (8.7)	18 (14.2)
Primary completed (n, %)	33 (70.2)	23 (67.7)	38 (82.6)	94 (74.0)
Secondary completed (n, %)	7 (15.0)	1 (2.9)	2 (4.4)	10 (7.9)
Higher education (n, %)	1 (2.1)	2 (5.9)	2 (4.4)	5 (3.1)
**Treatment supporters**	**36**	**34**	**37**	**107**
Female (n, %)	20 (55.6)	15 (44.1)	27 (73.0)	62 (57.9)
Median age (years) IQR	41	32	35	36
*Education*				
None (n, %)	4 (11.1)	7 (20.6)	12 (32.4)	23 (21.5)
Primary completed (n, %)	22 (61.1)	24 (70.6)	22 (59.5)	68 (63.5)
Secondary completed (n, %)	8 (22.2)	2 (5.9)	2 (5.4)	12 (11.2)
Higher education (n, %)	2 (5.6)	1 (2.9)	1 (2.7)	4 (3.7)
**Health workers**	**12**	**33**	**25**	**70**
Male (n, %)	10 (83.3)	17 (51.5)	16 (64.0)	43 (61.4)
*Qualification*				
Clinical Officer (n, %)	3 (23.1)	18 (56.3)	13 (52.0)	34 (48.6)
Nurse (n, %)	10 (76.9)	13 (40.6)	12 (48.0)	35 (50.0)
Unknown (n, %)	0 (0)	1 (3.1)	0 (0)	1 (1.4)

IQR, interquartile range.

Column percentages may not add up to 100% due to rounding.

### Freedom of choice for TB patients

In total, 32 patients (25.7%) were instructed to have their TB treatment either at home or to attend every day for health facility-based DOT, without being offered a choice. Furthermore, only 13.7% of those given a choice (13/95) confirmed that they were given enough time to make a decision (at least two weeks, as stipulated in the NTLP PCT guidelines). Some TB patients complained that the time given to make a decision was very short, as the following two statements testify:

*“Yes, they gave me options regarding the place of treatment but they did not give me enough time to think; they needed an answer the same day.”* (Patient 1)

*“Yes, I was given two days to make a choice regarding the place of treatment; I opted for home based-DOT.”* (Patient 2)

### Information on TB and its treatment given to patients and treatment supporters

**Figure 1:**
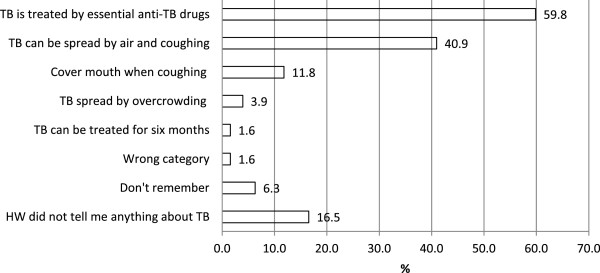
**Information about TB given to patients by health workers.** N = 127. Post-coding categories, multiple responses possible per respondent. HW, Health worker.

Among the 127 patients interviewed, 22.8% (n = 29) reported that they were not given any information about TB by health workers. However, information that TB is curable and can be treated with medicine was given to the majority of the patients. In total, 41% of patients (n = 52) were informed that TB is spread by air, but only 15 patients (11.8%) were told that TB transmission can be prevented by covering the mouth when coughing (Figure 1). Among treatment supporters, 81 (75.7%) were not informed how to monitor the patient’s adherence.

### TB drug side effects

**Figure 2:**
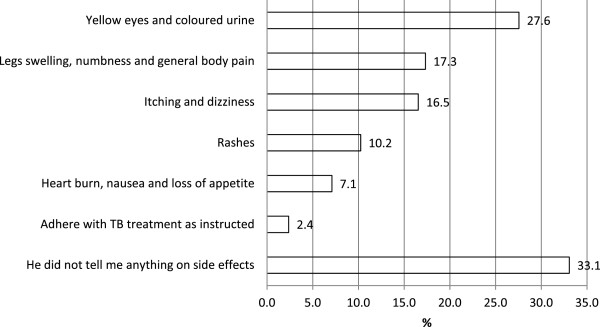
**TB drug side effects.** N = 127. Post-coding categories, multiple responses possible per respondent.

The majority of patients (66.9%, n = 85) confirmed that possible TB drug side effects were mentioned by health workers before the start of treatment. Side effects that patients could recall being described included yellow eyes and coloured urine (27.6%, n = 35), swelling of the feet (17.3%, n = 22), and itching and dizziness (16.5%, n = 21). According to the patients, there was no information given on what to do if these side effects occurred, including when to return to the health facility (Figure 2).

### Factors positively influencing treatment completion

All 70 health workers were asked what factors favour successful completion of TB treatment by their patients. The most common factor reported was proper health education of patients (45.7%; 32/70), followed by patients having a good and reliable treatment supporter (25.7%; 18/70) and understanding the treatment plan (22.9%; 16/70). Other factors mentioned included fear of death (14.3%; 10/70) and a smaller number of pills (4.3%; 3/70).

*“Some patients complete treatment because they want to be cured.”* (Health worker 1)

*“They follow health workers’ instructions.”* (Health worker 2)

### Health workers assessments of patient adherence

The majority of health workers (68.6%; 48/70) said that they checked the blister packs that were returned by home-based patients, to verify the drugs had been taken as required. Checking the ticks on patients’ treatment identification cards (20.0%; 14/70) and medical prognosis (8.6%; 6/70) were mentioned infrequently as additional assessments.

*“Patients’ cards confirm adherence to drug administration.”* (Health worker 3)

*“Yes, supporters bring empty blister packets and filled patients’ cards.”* (Health worker 4)

## Discussion

Our study shows that some key elements of PCT approach are implemented inadequately by health workers. This may compromise patient adherence and ultimately treatment outcomes. Still a quarter of the patients were not given a choice by health workers concerning the place of treatment. If health facility-based treatment is forced upon the patients adherence may be compromised if the distance between patients’ residences and the health facility is too big [14]. Furthermore, it precludes health workers from engaging in other important daily tasks [15-17]. However, if patients are forced to follow home-based DOT, this may also result in poor treatment adherence especially when they do not have a reliable treatment supporter. The fact that only about 14% of those who were given the choice felt they had enough time to think of the two options and identify a suitable treatment supporter may lead to a wrong choice with regard to the treatment supporter, or even result in health facility DOT although they prefer home based DOT.

The study revealed that patients and treatment supporters do not seem to be properly informed about TB by health workers. This is of particular relevance since health workers identified health education as a pivotal factor in promoting treatment completion. Adequate knowledge about the spread of TB during treatment may prevent unnecessary social isolation, while understanding the duration of treatment gives the patient a better perspective about his/her abilities in the near future [18]. Taken together, this information will guide the patient and supporter through the period of TB treatment and without it maintaining adherence to therapy may be challenging.

Providing information on the potential side effects of TB drugs did not appear to be a priority for a significant number of healthcare providers, since approximately one third of patients did not receive any information on side effects. It has, however, been documented that drug side effects negatively influence adherence to treatment [18] and patients who experience such events are more likely to discontinue treatment [19].

Although health workers did not adequately instruct patients and supporters, they nevertheless identified the importance of health education and reliable treatment supporters as factors which positively influence patient adherence and successful treatment outcome. Additional health education given to newly diagnosed TB patients may motivate them to complete TB treatment [20], and the support of family members appears to have a strong positive influence on patient adherence to treatment [9].

Checking returned blister packs from home-based patients was the primary method by which health workers assessed patient adherence. This corresponds with NTLP guidelines for treatment adherence. However, WHO recommends that counting empty blister packs is not an acceptable method for determining adherence, especially in the case of multidrug-resistant treatment [21].

Two factors appear crucial for improving improve health workers’ performance and ultimately the effectiveness of the PCT delivery strategy. Firstly, health workers should be re-trained and made aware of the key elements of PCT. Secondly, regular supportive supervision and mentoring of health workers by the district health authorities are indispensable for ensuring the quality of TB care. The scale-up of PCT through dissemination of new guidelines without any training shows that this is an inadequate strategy. With guidelines not being followed, and health workers not being supervised, the initial positive PCT results may be compromised, leading to *de facto* TB self-treatment, higher rates of non-adherence and an increase in the prevalence of drug resistance.

The NTLP of Tanzania has welcomed this evaluation and identified the need for health worker training. In collaboration with other partners, it has produced an educational film for health workers, patients and treatment supporters that provides a simplified explanation of all the main steps and elements of PCT. The video is distributed to every hospital and health centre with TB services and functioning DVD/TV devices. When distribution of this educational tool is accompanied by supportive supervision of health workers by well-trained Regional and District TB and Leprosy Coordinators, PCT can be implemented successfully in Tanzania.

The presented study has limitations. The dominant local languages sometimes had to be used in the rural districts of Kahama and Mufindi (Kisukuma and Kihehe, respectively) where respondents were not able to express themselves fluently in Kiswahili, with the risk of possible information bias from these respondents. However, health workers who were not involved in TB treatment undertook the interviews, with care workers or community members serving as interpreters, and back-translation was undertaken immediately to reduce the potential for bias.

Although the data were collected in 2007, a year after scale-up of the PCT approach in Tanzania, it is unlikely that the findings have been superseded. Since the scale-up, there have been no changes with respect to treatment allocation or observation. If anything, the need for training and regular supportive supervision is now more urgent than ever.

## Conclusion

This study identified several weaknesses and programmatic challenges in the implementation of the PCT strategy for TB treatment in Tanzania. The provision of choice of treatment location, information on treatment, and guidance for treatment supporters need to be improved. There is a requirement for regular re-training of health workers with effective supportive supervision if successful implementation of the PCT approach is to be sustained.

## Competing interests

None of the authors declares a competing interest. The study was funded by the Novartis Foundation for Sustainable Development (NFSD). AS was a full time employee of the NFSD throughout the study period.

## Authors’ contributions

AM was involved in the study design, carried out field work, undertook the analysis and interpretation of data, and drafted the manuscript. NR was involved in the study design, carried out field work and revised the manuscript. MA was involved in the study design, carried out field work and revised the manuscript. EG revised the manuscript. SE was involved in the study design and revised the manuscript. FL was involved in the study design, carried out field work and commented on the manuscript. AS revised the manuscript. FvL was involved in the study design and the interpretation of data, and revised the manuscript. All authors read and approved the final manuscript.

## Pre-publication history

The pre-publication history for this paper can be accessed here:

http://www.biomedcentral.com/1472-6963/13/101/prepub

## References

[B1] Global Tuberculosis Control: Epidemiology S, Financing 2009 Geneva: Switzerland World Health Organization http://whqlibdoc.who.int/publications/2009/9789241563802_eng_doc.pdf

[B2] Broekmans JF Tuberculosis and HIV-infection in developing countries Trop Geogr Med 1991 43 S13 S21 1687768

[B3] Nuhu A AIDS blamed for increase in Tanzanian tuberculosis cases J Int Assoc Physicians AIDS Care 1995 1 27 11362233

[B4] Range N Ipuge YA O’Brien RJ Egwaga SM Mfinanga SG Chonde TM Mukadi YD Borgdorff MW Trend in HIV prevalence among tuberculosis patients in Tanzania, 1991–1998 Int J Tuberc Lung Dis 2001 5 405 412 11336270

[B5] van Cleeff MR Chum HJ The proportion of tuberculosis cases in Tanzania attributable to human immunodeficiency virus Int J Epidemiol 1995 24 637 642 10.1093/ije/24.3.637 7672908

[B6] Jindani A Nunn AJ Enarson DA Two 8-month regimens of chemotherapy for treatment of newly diagnosed pulmonary tuberculosis: international multicentre randomised trial Lancet 2004 364 1244 1251 10.1016/S0140-6736(04)17141-9 15464185

[B7] Egwaga S Range N Lwilla F Mkopi A Barongo V Mtenga S Mshinda H Cobelens F Haag V van Leth F Grewal P Assessment of patient preference in allocation and observation of anti-tuberculosis medication in three districts in Tanzania Patient Prefer Adherence 2008 2 1 6 19920938 PMC2770399

[B8] Egwaga S Mkopi A Range N Haag-Arbenz V Baraka A Grewal P Cobelens F Mshinda H Lwilla F van Leth F Patient-centred tuberculosis treatment delivery under programmatic conditions in Tanzania: a cohort study BMC Med 2009 7 80 10.1186/1741-7015-7-80 20025724 PMC2801503

[B9] Newell JN Baral SC Pande SB Bam DS Malla P Family-member DOTS and community DOTS for tuberculosis control in Nepal: cluster-randomised controlled trial Lancet 2006 367 903 909 10.1016/S0140-6736(06)68380-3 16546538

[B10] Chaulk CP Kazandjian VA Directly observed therapy for treatment completion of pulmonary tuberculosis: consensus statement of the public health tuberculosis guidelines panel JAMA 1998 279 943 948 10.1001/jama.279.12.943 9544769

[B11] Brust JC Shah NS Scott M Chaiyachati K Lygizos M van der Merwe TL Bamber S Radebe Z Loveday M Moll AP Integrated, home-based treatment for MDR-TB and HIV in rural South Africa: an alternate model of care Int J Tuberc Lung Dis 2012 16 998 1004 10.5588/ijtld.11.0713 22668560 PMC3390442

[B12] NTLP How to Provide Patient Centred TB Treatment: A guide for health workers 2005 Dar es Salaam: Ministry of Health and Social Welfare

[B13] Pope C Ziebland S Mays N Qualitative research in health care. Analysing qualitative data BMJ 2000 320 114 116 10.1136/bmj.320.7227.114 10625273 PMC1117368

[B14] Munro SA Lewin SA Smith HJ Engel ME Fretheim A Volmink J Patient adherence to tuberculosis treatment: a systematic review of qualitative research PLoS Med 2007 4 e238 10.1371/journal.pmed.0040238 17676945 PMC1925126

[B15] Sanou A Dembele M Theobald S Macq J Access and adhering to tuberculosis treatment: barriers faced by patients and communities in Burkina Faso Int J Tuberc Lung Dis 2004 8 1479 1483 15636495

[B16] Singh V Porter JDH Ogden JA Sarin R Sharma PP Arora VK Jain RC TB control, poverty and vulnerability in Delhi India Trop Med Int Health 2002 7 693 700 10.1046/j.1365-3156.2002.00909.x 12167096

[B17] Advocacy casmfTcAGTDK, ATTITUDE AND PRACTICE SURVEYS 2008 Geneva, Switzerland: World Health Organization http://www.stoptb.org/assets/documents/resources/publications/acsm/ACSM_KAP%20GUIDE.pdf

[B18] Jaiswal A Singh V Ogden JA Porter JD Sharma PP Sarin R Arora VK Jain RC Adherence to tuberculosis treatment: lessons from the urban setting of Delhi, India Trop Med Int Health 2003 8 625 633 10.1046/j.1365-3156.2003.01061.x 12828545

[B19] Frieden TR Sbarbaro JA Promoting adherence to treatment for tuberculosis: the importance of direct observation Bull World Health Organ 2007 85 407 409 10.2471/BLT.06.038927 17639230 PMC2636637

[B20] Dick J Van der Walt H Hoogendoorn L Tobias B Development of a health education booklet to enhance adherence to tuberculosis treatment Tuber Lung Dis 1996 77 173 177 10.1016/S0962-8479(96)90034-9 8762854

[B21] Adherence to long term therapies: evidence for action 2003 Geneva Switzerland: World Health Organization http://www.who.int/chp/knowledge/publications/adherence_introduction.pdf

